# Effects of Conical Positive Expiratory Pressure Mask Application During Exercise Training on Pulmonary Rehabilitation Outcomes in Moderate to Severe COPD Cases: A Randomized Controlled Trial

**DOI:** 10.1155/pm/8828987

**Published:** 2025-10-31

**Authors:** Chulee Ubolsakka-Jones, David Arthur Jones, Malipron Pukdeechat, Watchara Boonsawat, Wilaiwan Khrisanapant, Pornanan Domthong, Seksan Chaisuksant, Piyaraid Dongkhanti, Aung Aung Nwe, Chatchai Phimphasak

**Affiliations:** ^1^School of Physical Therapy, Faculty of Associated Medical Sciences, Khon Kaen University, Mueang Khon Kaen, Khon Kaen Province, Thailand; ^2^Innovation to Improve Cardiopulmonary and Physical Performances Research Group, Khon Kaen University, Mueang Khon Kaen, Khon Kaen Province, Thailand; ^3^Division of Health Science, School of Health Sciences, Manchester Metropolitan University, Manchester, UK; ^4^Faculty of Sport and Health Sciences, Thailand National Sports University Mahasarakham Campus, Mueang Mahasarakham, Mahasarakham Province, Thailand; ^5^Division of Respiratory Medicine, Department of Internal Medicine, Faculty of Medicine, Khon Kaen University, Mueang Khon Kaen, Khon Kaen Province, Thailand; ^6^Department of Physiology, Faculty of Medicine, Khon Kaen University, Mueang Khon Kaen, Khon Kaen Province, Thailand; ^7^Department of Internal Medicine, Khon Kaen Hospital, Mueang Khon Kaen, Khon Kaen Province, Thailand; ^8^Department of Physical Therapy, Atsamat Hospital, Atsamat, Roi Et Province, Thailand

## Abstract

**Background:**

The use of positive expiratory pressure (PEP), which includes conical-PEP breathing, has been proposed for use during exercise among patients with chronic obstructive pulmonary disease (COPD) to reduce dynamic hyperinflation (DH) and improve exercise capacity. However, evidence on the effects of exercise training with conical-PEP for pulmonary rehabilitation (PR) remains limited. This study was conducted to evaluate the aforementioned effects on exercise capacity, DH, and quality of life among patients with moderate to very severe COPD.

**Methods:**

Forty-two patients with moderate to very severe COPD were assigned to a home-based PR program. They were then randomly allocated to exercise training with conical-PEP (*n* = 21, age 64.5 ± 6.8 years) or without conical-PEP (control group, *n* = 21, age 67.2 ± 8.0 years) for 8–10 weeks. The outcomes of the 6-min walk distance (6MWD), the endurance spot marching test (ESMT) for endurance time, an inspiratory capacity (IC) test to assess DH, the transition dyspnea index (TDI), St. George's Respiratory Questionnaire (SGRQ), and the COPD Assessment Test (CAT) were recorded at baseline and at the program's end (post-PR).

**Results:**

There were no significant differences in 6MWD (*p* = 0.116) or ESMT endurance time (*p* = 0.247) between the conical-PEP and control groups at post-PR. Compared to baseline, the post-PR measurements showed a significant reduction in end-exercise IC in the control group (*Δ* −0.08 L, 95% CI: −0.16 to −0.01 L, *p* = 0.033) but no significant reduction in the conical-PEP group (*Δ* −0.07 L, 95% CI: −0.19 to 0.05 L, *p* = 0.193). No significant differences were found between the groups at post-PR in terms of TDI (*p* = 0.277), SGRQ (*p* = 0.687), or CAT (*p* = 0.704) scores.

**Conclusion:**

The addition of conical-PEP during exercise training for PR in COPD did not provide significant benefits over exercise training without conical-PEP. Further research is warranted.

## 1. Introduction

Chronic obstructive pulmonary disease (COPD) is one of the most prevalent diseases worldwide and is characterized by chronic inflammation, small airway dysfunction, and lung parenchymal destruction. The resulting airway instability and reduced or lost elastic recoil lead to airflow limitation and hyperinflation. These pathological changes ultimately cause dyspnea, a decline in exercise capacity, and decreased patient quality of life [1].

Pulmonary rehabilitation (PR) is a well-established intervention recommended for patients with COPD. PR programs typically include a range of interventions with exercise training as the core component, as this has been shown to increase exercise capacity, reduce dyspnea, alleviate anxiety and depression, improve muscle strength, enhance health-related quality of life (HRQoL), and reduce the frequency of exacerbations and hospital admissions and the length of hospital stays [2–7]. As a result of airway instability and loss of elastic recoil, patients with COPD tend to experience premature airway closure due to the distal movement of the equal pressure point (EPP) during forced expiration (e.g., during exercise). This leads to dynamic hyperinflation (DH) and a reduction in the operational lung volume. DH is a key factor contributing to dyspnea. Patients with COPD often experience rapidly worsening dyspnea, which limits their exercise duration [8, 9], and shortened exercise times may not be sufficient to effectively improve physical performance.

To address this gap, the application of positive expiratory pressure (PEP) during exercise has gained attention. Theoretically, applying PEP increases airway pressure and shifts the EPP back to the proximal airways, thereby reducing premature airway closure and limiting DH [10]. Several studies have examined the effects of PEP on exercise capacity and DH in patients with COPD. Russo et al. reported significant improvements in exercise capacity with threshold PEP levels of 1 and 10 cmH2O [11]. Similarly, Nicolini et al. reported that the exercise capacities of COPD patients increased upon using a threshold PEP of 5 cmH2O [12]. In contrast, Wibmer et al. observed a reduction in exercise capacity with flow-dependent PEP (10–20 cmH2O) administered via a nasal mask, compared to controls [13]. Gass et al. too found that exercise capacity decreased for moderate to severe COPD patients upon using threshold PEP levels of 5 and 10 cmH2O during cycling [14]. These conflicting findings may be attributed to differences in devices and techniques. Notably, these studies focused only on the immediate effects of PEP in single exercise sessions.

Conical-PEP, a type of flow-dependent PEP, has a key advantage over flow-independent PEP: it does not require the build-up and maintenance of pressure throughout expiration and thus prevents any interruption of expiratory flow [15]. Ubolsakka-Jones et al. and Kosura et al. demonstrated that conical-PEP provided via an oronasal mask improved exercise capacity and DH during a single session in COPD [16, 17]. The aim of the present study was to investigate the long-term effects of conical-PEP application via an oronasal mask during PR on the exercise capacity, DH, lung function, dyspnea, and HRQoL of patients with COPD.

## 2. Methods

### 2.1. Participants

A randomized controlled trial was conducted to evaluate the effects of conical-PEP in this study. Ethical approval was granted by the Human Research Ethics Committee of Khon Kaen University (HE591337) and Khon Kaen Hospital (KE 60132), and the study was registered with the clinical trial registry. Informed consent was obtained from all eligible participants prior to the study's commencement. Participants were recruited from Srinagarind Hospital, Khon Kaen Hospital, and Phra Yuen Hospital in Thailand between October 2016 and December 2017. They performed a home-based PR program, and the outcomes were assessed at Phra Yuen Hospital or the School of Physical Therapy, Faculty of Associated Medical Sciences, Khon Kaen University, Thailand.

### 2.2. Eligible Criteria

The inclusion criteria were physician-diagnosed moderate to very severe COPD, postbronchodilator FEV_1_/FVC < 0.7, and age between 40 and 80 years. The exclusion criteria included exacerbation and medication change within the past month, the use of home oxygen therapy, musculoskeletal conditions that affected exercise, the use of an assistive device for walking, and heart diseases such as coronary artery disease or valvular heart disease.

### 2.3. Sample Size Calculation

Based on previous research data on exercise capacity after a PR program that included pursed-lip breathing (PEP breathing without a device) [18], a sample size of 18 per group was calculated using G∗power (3.1.9.7), with an alpha of 0.05 and a power of 95%. Upon accounting for a 15% dropout rate, 21 participants were enrolled in each study group, resulting in a total of 42 participants.

### 2.4. Randomization

Stratified block randomization (stratified by COPD GOLD Stage II or GOLD Stages II and IV with block sizes of 6, 4, and 2) was manually performed by M.P., the third author, to allocate the participants to either the conical-PEP group or the control group in a 1:1 ratio. The assignments (conical-PEP and control) were sealed in numbered envelopes corresponding to specific blocks. Participant enrolment was handled by M.P., the third author, while the group assignments were performed by C.P., the tenth author.

### 2.5. Home-Based PR Program

All participants underwent a home-based PR program for 8–10 weeks. The participants performed a self-paced spot marching exercise; individualized step rates were prescribed based on each participant's performance during an endurance spot marching test (ESMT) [17]. The subjects were instructed to perform spot marching in the morning or evening, as per their preference, until they obtained a dyspnea level of 3–4 out of 10 on the modified Borg scale (rating of perceived breathlessness, RPB). They then had to rest until their RPB dropped to less than 1 out of 10 and subsequently resume spot marching following the same protocol. Exercise progression occurred naturally, as improved tolerance delayed dyspnea onset, allowing for longer exercise durations to reach the target RPB levels. The recommended total exercise time was 30–60 min per day, 3–5 days per week. Exercises were to be performed 15–20 min after routine bronchodilator use. Stretching exercises for the shoulders, trunk, and lower limbs were recommended for 5–10 min before and after each spot marching session.

During their first visit after recruitment, all participants were provided with instructions for breathing strategies that they could use to manage dyspnea during activities of daily living, following exercise, and after coughing. Patients with secretion-related problems were also taught the active cycle of breathing technique. Further educational content included information on COPD pathophysiology, basic nutrition, smoking cessation, dyspnea management, the appropriate use of emergency bronchodilators, and correct inhaler techniques. Usual medications were continued throughout the study.

Preparation and termination instructions for the exercise were provided to all participants on a printed pamphlet. Each participant was visited at home once during the first 4 weeks of the PR program to review and reinforce adherence. They were also contacted by telephone at least once to encourage continued exercise.

### 2.6. Intervention

When performing the self-paced spot marching exercise, the conical-PEP group used a conical-PEP device equipped with a nonrebreathing face mask that covered both the mouth and nose [17]. The conical-PEP resistors were 1 cm in length, with 5, 6, and 7-mm orifices, and they provided a PEP of approximately 4.8 ± 2.5 cmH2O (range: 3.0–14.5 cmH2O). The resistor sizes were selected to match the expected airflow during the exercise [17]. The participants were instructed to prolong their expiration through the conical-PEP device during the exercise.

### 2.7. Control

The control group performed the same spot marching exercise but without the conical-PEP device.

### 2.8. Exercise Characteristics and Perception

Participants in both groups were provided with a logbook to document their exercise sessions throughout the program. They were instructed to record the number of sessions per day, the duration of each session, a perceived breathlessness rating at the end of each session, the reason for stopping exercise, and, for the conical-PEP group, whether the conical-PEP mask was used during the sessions.

### 2.9. Outcome Measures

Before the PR program commenced, the participants' steps per day and activity times were measured for five consecutive days using a pedometer (Yamax Digital Walker CW700) to determine the physical activity levels of the demographic. Assessments of exercise capacity, DH, lung function, dyspnea, and HRQoL were conducted at baseline and at the end of the PR program.

#### 2.9.1. Exercise Capacity

Exercise capacity was assessed using the 6-min walk test (6MWT) [19], and endurance time was measured using the ESMT [17]. The procedure for the ESMT was as follows: The participants first performed the incremental spot marching test (ISMT) by marching in place with alternating arm (90°) and leg (hip flexion 70°) movements. The exercise began at a step rate of 50–70 steps/min, with increments of 5–10 steps/min every 2 min until a rate of 120 steps/min was reached or volitional fatigue occurred. The peak step rate achieved in the ISMT, with a RPB ≥ 2 and a correct movement pattern, was used as the constant step rate for the ESMT. Participants marched in place at this constant step rate until reaching volitional fatigue or for a maximum of 25 min. Physiological responses, namely, heart rate, blood pressure, end-tidal CO_2_, oxygen saturation (SpO_2_), RPB, respiratory rate, and leg fatigue (assessed using the modified Borg scale), were recorded before and at the end of the exercise and during a 10-min recovery period for both the 6MWD and ESMT. Percentage of predicted age-related maximum heart rate (%HRmax) was calculated from 206.9 − (0.69∗age) [20].

#### 2.9.2. DH

DH was assessed by measuring the inspiratory capacity (IC) change before and immediately after (within 30 s) the ESMT and after a 10-min recovery period. A Pneumotach KOKO spirometer (United States) was used to measure DH in this study, in line with the ATS/ERS recommendations for inspiratory IC.

#### 2.9.3. Lung Function

Forced and slow vital capacity maneuvers were performed using a pneumotach KOKO spirometer (United States), following the ATS/ERS task force recommendations for the standardization of lung function testing [21]. Maximum inspiratory pressure (MIP) from residual volume (RV) and maximum expiratory pressure (MEP) from total lung capacity (TLC) were measured using a MicroRPM respiratory pressure meter. MIP and MEP were measured 3–6 times, with the highest values taken for analysis.

#### 2.9.4. Dyspnea

Dyspnea symptoms were assessed using the transition dyspnea index (TDI), which measures changes in dyspnea severity compared to the baseline dyspnea index (BDI).

#### 2.9.5. HRQoL

St. George's Respiratory Questionnaire (SGRQ) and the COPD Assessment Test (CAT) were used to measure HRQoL.

### 2.10. Data Analysis

Descriptive statistics, including mean and standard deviation (SD) values, medians, and interquartile ranges (IQRs), were used to report each subject's characteristics and cardiopulmonary responses to the exercise tests. The Shapiro–Wilk test was used to test the data for distribution normality. Paired *t*-tests or the Wilcoxon signed-rank test were used to compare the parameters within a group. Independent *t*-tests, the Mann–Whitney *U* test, two-sample tests for proportion, Pearson's chi^2^, or analysis of covariance (ANCOVA) with post hoc testing was used to compare differences between groups. A one-way repeated measures ANOVA with the Bonferroni correction was used to compare IC responses at the pre-exercise, end-exercise, and recovery time points for each ESMT. A one-way ANOVA with the Bonferroni correction was used to compare the baseline and post-PR IC measurements between groups at the pre-exercise, end-exercise, and recovery time points. Pearson's correlation was used to analyze the relationship between exercise volume and exercise capacity. An intention-to-treat analysis using the hot deck imputation method was applied for participants who were lost to follow-up. The *p* value was set at 0.05. Stata Version 10 (StataCorp, College Station, Texas) was used for the analysis.

## 3. Results

**Figure 1:**
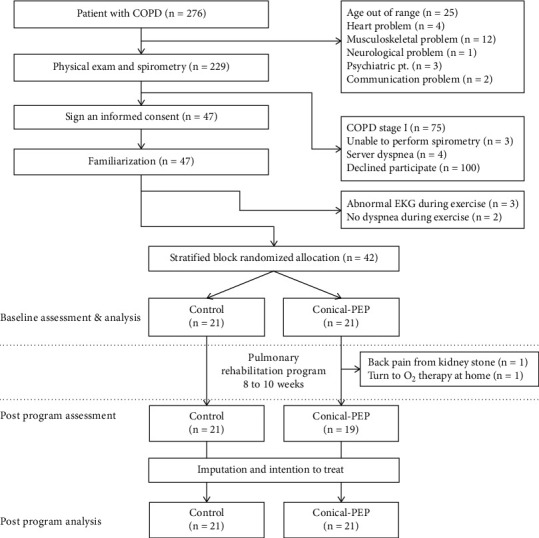
Participant flow diagram.

The participants were enrolled in and completed the study between October 2016 and December 2017. A total of 459 patients were screened using spirometry, 147 met the inclusion and exclusion criteria, and 47 subjects provided informed consent. Among them, 42 patients with moderate to very severe COPD were randomly assigned to either the control group or the conical-PEP group and completed the study (see Figure 1). The characteristics of the participants are presented in Table 1; no significant differences were observed between the groups before the PR program.

Table 2 presents the exercise characteristics of the participants in the home-based PR program. There were no significant differences between the groups in terms of exercise time per session and other exercise parameters.

### 3.1. Exercise Capacity

**Figure 2:**
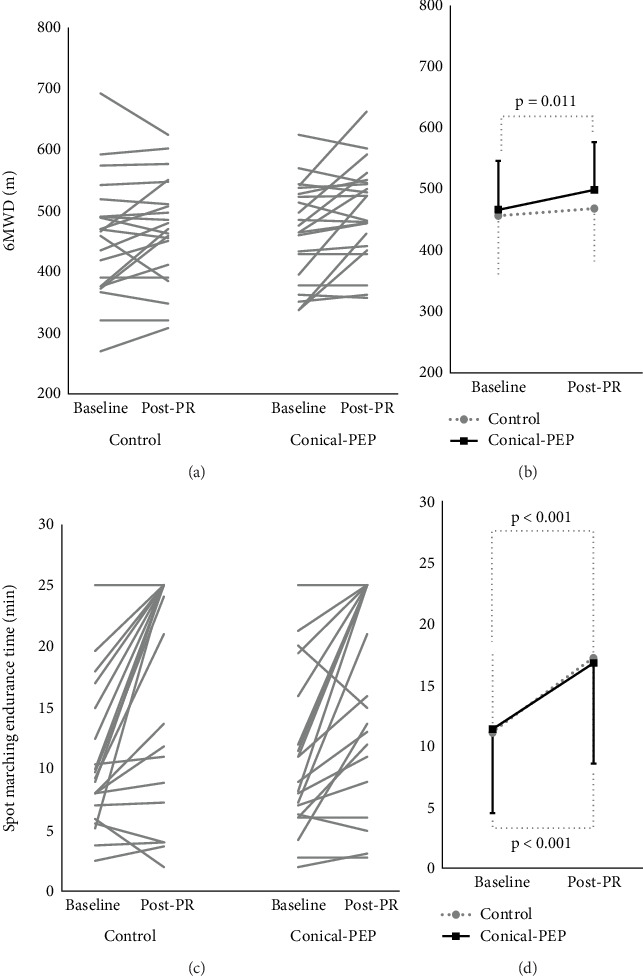
Exercise capacity; 6-min walk distance ((a) individual plot, (b) mean ± SD) and spot marching endurance time ((c) individual plot, (d) mean ± SD) at baseline and post-PR. The *p* value represents the significant within-group difference obtained using a paired *t*-test.

Table 3 and Figure 2 present the 6MWD and ESMT endurance times. There was no significant difference in 6MWD between the control and conical-PEP groups at baseline and post-PR. Compared to baseline, the conical-PEP group showed a significant improvement in 6MWD (*Δ*32.2 m, 95% CI: 8.3–56.2 m, *p* = 0.011), but no significant difference was observed in the control group (*Δ*12.3 m, 95% CI: −7.6 to 31.8 m, *p* = 0.205) (Figure 2b). Regarding ESMT endurance time, there was no significant difference between the two groups' measurements at baseline and post-PR. Intragroup analyses revealed significant improvements in ESMT endurance time, with a mean change of *Δ*6.0 min in the conical-PEP group (95% CI: 3.0–9.1 min, *p* < 0.001) and *Δ*5.4 min in the control group (95% CI: 2.6–8.2 min, *p* < 0.001) (Figure 2d).

**Figure 3:**
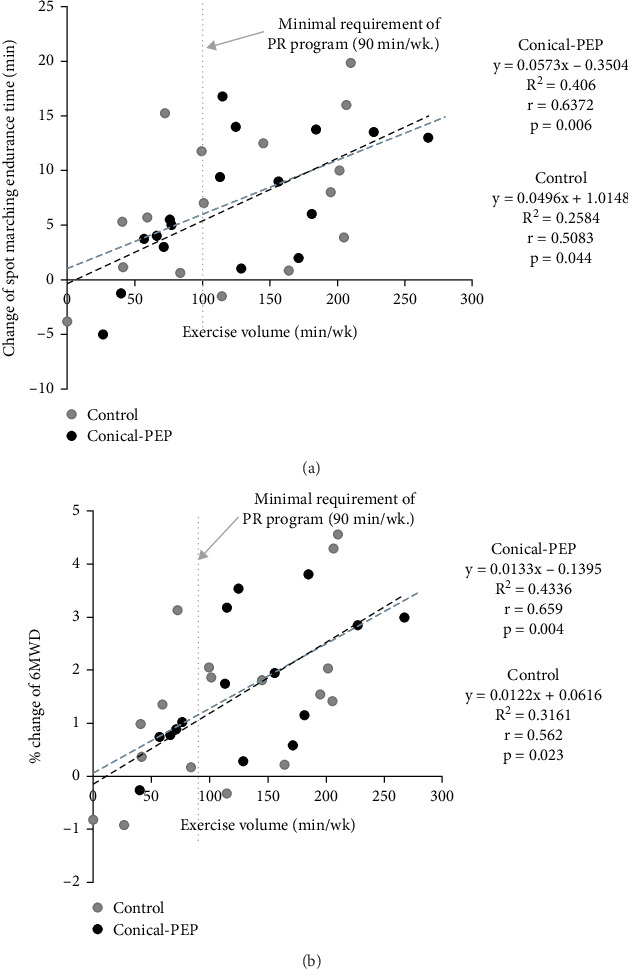
The relationship between the exercise parameters at post-PR; (a) exercise volume versus change in spot marching endurance time, (b) exercise volume versus percentage change in 6MWD.

The relationship between changes in exercise volume and improvements in exercise capacity, measured based on the 6MWD and ESMT endurance times, is shown in Figure 3. The findings showed a positive correlation between the change in exercise volume and exercise capacity for both the 6MWD (*r* = 0.562, *p* = 0.023) and ESMT endurance times (*r* = 0.508, *p* = 0.044).

### 3.2. DH

**Figure 4:**
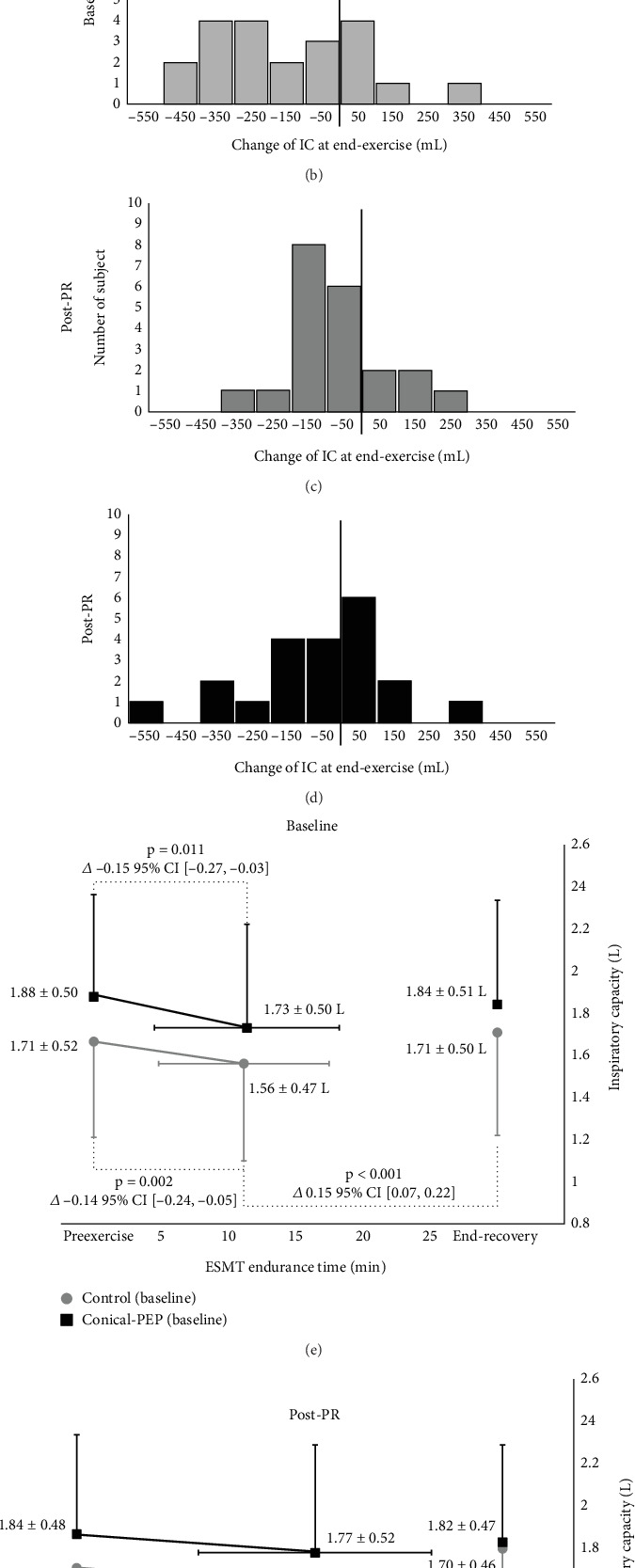
IC change at the end of the exercise based on the endurance spot marching test. (a–d) The distribution of IC change. (a, c) Control group at baseline and post-PR; (b, d) conical-PEP group at baseline and post-PR; IC change plot against ESMT endurance time at (e) baseline and (f) post-PR.

End-exercise IC significantly reduced in both groups at baseline (Figure 4e). At post-PR, a significant reduction in end-exercise IC was observed in the control group (*Δ* −0.08 L, 95% CI: −0.16 to −0.01 L, *p* = 0.033) but not in the conical-PEP group (*Δ* −0.07 L, 95% CI: −0.19 to 0.05 L, *p* = 0.193) (Figure 4f). Nonsignificant differences in baseline and post-PR IC were observed between the groups at the pre-exercise, end-exercise, and recovery time points.

### 3.3. Dyspnea

**Figure 5:**
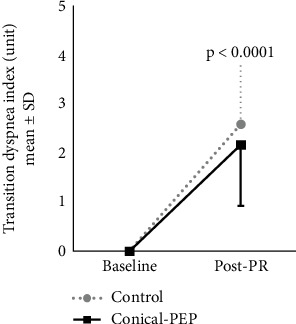
Transition dyspnea index at baseline and post-PR (8 weeks); *p* value indicates a significant difference with baseline for both groups. Data are presented as mean ± SD values.

The BDI scores were 8.8 ± 1.2 for the control group and 8.2 ± 1.6 for the conical-PEP group. TDI increased significantly by 2.6 points in the control group (95% CI: 2.1–3.2, *p* < 0.0001) and 2.2 points in the conical-PEP group (95% CI: 1.6–2.8, *p* < 0.0001) (Figure 5). No significant difference in TDI was observed between the two groups at post-PR (*p* = 0.277).

### 3.4. Quality of Life

**Figure 6:**
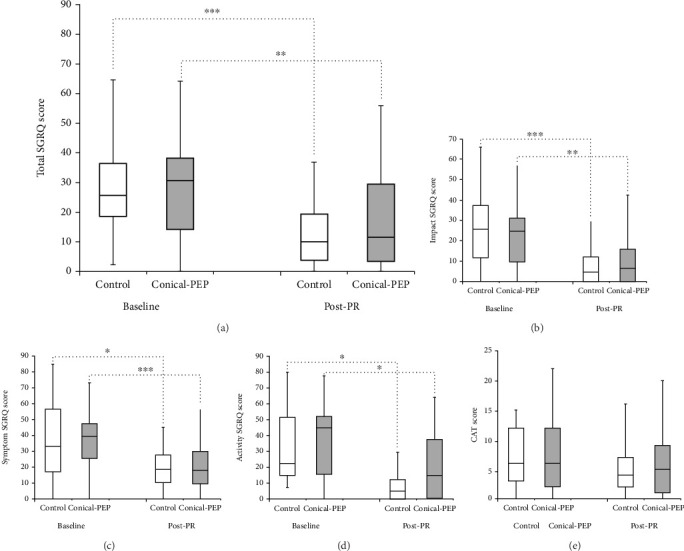
Box plot of HRQoL at baseline and post-PR. (a) Total SGRQ score, (b) symptom SGRQ score, (c) activity SGRQ score, (d) impact SGRQ score, and (e) CAT score; ⁣^∗^significant with baseline ⁣^∗^*p* < 0.05, ⁣^∗∗^*p* < 0.01, and ⁣^∗∗∗^*p* < 0.001 for the Wilcoxon signed-rank test.

Regarding the SGRQ (Table 4), no significant differences in total score were found between the two groups at baseline and at post-PR. However, the within-group analysis revealed significant improvements in both groups' SGRQ scores at post-PR compared to baseline. For the CAT score, no statistically significant differences were observed between the control and conical-PEP groups at baseline or post-PR (Figure 6).

### 3.5. Physiological Responses at the End of 6MWT and ESMT

The pre-exercise and postexercise physiological responses at baseline and post-PR for 6MWT and ESMT are shown in Table S1A and S1B. In the post-PR 6MWT, pre-exercise and end-exercise heart rate and %HRmax measurements showed no significant differences between the groups. The end-exercise RPB scores also showed no significant differences between the groups.

In the post-PR ESMT, pre-exercise and end-exercise heart rate and %HRmax measurements showed no significant differences between the groups. The end-exercise RPB scores also showed no significant differences between the groups.

### 3.6. Lung Function

Regarding lung function parameters, no significant differences were observed between the two groups at baseline. A within-group analysis showed significant improvements in FEV1, FEV1% predicted, FEF25%–75% predicted, peak expiratory flow (PEF), and PEF% predicted in the control group. In the conical-PEP group, significant differences were found in the PEF and PEF% predicted values compared to baseline. However, no significant differences in lung function were observed between the two groups at the end of the program (Table 5).

## 4. Discussion

To the best of our knowledge, this was the first study to investigate the training effects of conical-PEP in a PR program. Our findings revealed that exercise training, with or without conical-PEP, improved clinical outcomes such as exercise capacity, dyspnea, and HRQoL, but no statistically significant differences were observed between the control and conical-PEP groups. However, exercise training with conical-PEP showed a tendency to delay the development of DH.

Regarding exercise capacity, both groups' ESMT improved significantly after the program, and no significant difference was observed between the two groups. Regarding 6MWD, significant improvements were observed at the end of the PR program in the conical-PEP group compared to baseline, whereas the control group's changes were not statistically significant. However, no statistically significant difference was found between the two groups. The conical-PEP group showed a 32.2 m improvement in 6MWD from the baseline, which exceeded the minimal clinically important difference (MCID) [22], whereas the control group showed a 12.3 m increase from the baseline that did not reach the MCID. It is difficult to directly compare the results of this study with those of previous studies that used PEP to enhance exercise capacity, as previous studies typically involved very short-term interventions, such as single sessions. For instance, Nicolini et al. demonstrated an improvement in 6MWD in patients with moderate to severe COPD when a 5 cmH2O spring load PEP was used during a single session, compared to a control group [12]. Further, Ubolsakka-Jones et al. and Kosura et al. reported improved endurance times among patients with moderate to severe COPD when they performed the ESMT with a conical-PEP (around 5 cmH2O) in a single session, compared to a control group [16, 17]. The results of these short-term studies are inconsistent with those of the present study. One possible reason for the differences is that a conical-PEP was used during the training phase of the PR program in the present study, and 6MWT and ESMT were assessed without conical-PEP. Therefore, the findings indicate the training effects of conical-PEP, in contrast to previous studies that evaluated the immediate effects of PEP during a single exercise session.

Our study revealed no significant differences between the groups in terms of exercise time per session and other exercise parameters (Table 2). This contrasts with our previous findings of Ubolsakka et al. and Kosura et al., which showed that PEP could increase exercise duration during a single supervised session [16, 17]. However, that study involved direct supervision, whereas the present trial involved a home-based program. The lack of supervision may have contributed to the discrepancy, as participants may be capable of exercising harder or longer but do not necessarily do so without reinforcement.

The training effects of PEP on exercise capacity in COPD cases remain underexplored, although pursed-lip breathing, a spontaneous PEP that generates approximately 5 cmH2O, has shown some benefits. Casciari et al. examined the impact of a 6-week breathing retraining program, including pursed-lip breathing during daily activities and exercise, on patients with severe COPD and reported improvements in exercise capacity based on oxygen consumption [18]. Similarly, Xu et al. found that exercise capacity, as measured by the 6MWD, increased after the use of PEP at 5 cmH2O for 4 h per day for 2 months during daily activities [23]. In our study, around 5 cmH2O of PEP was applied using a conical-PEP, but the focus was specifically on its application during exercise rather than daily activities.

No significant differences in lung function were found between the conical-PEP and control groups, nor were there any clinically meaningful changes compared to baseline in either group. These outcomes were expected, considering that the pathology of COPD is generally considered irreversible. The findings align with a systematic review and meta-analysis by Chen et al., which also revealed no significant changes in lung function following exercise rehabilitation in patients with COPD [24].

Our results showed an improvement in dyspnea in both groups after 8 weeks of PR, as reflected by the BDI and TDI scores. The control and conical-PEP groups improved by 2.6 and 2.2 points, respectively, both exceeding the MCID of 1 point [25]. The TDI improvement in the present study was higher than the mean change of 1–1.5 points after 8 weeks of PR reported by Casaburi et al. [26] and the mean change of 0.6–0.8 points after 6 weeks of PR in Sassi-Dambron et al.'s study [27]. Although the outcomes improved following the PR program, no significant difference was observed between the two groups. One possible explanation for the lack of difference between the groups is that conical-PEP was applied only during self-paced spot marching exercises and not during other activities. Additionally, both groups were educated and trained in using the pursed-lip breathing technique during activities, which could have contributed to the similarity in outcomes between the two groups. Therefore, exercising with conical-PEP may improve exercise capacity, but it might not have a great effect on dyspnea compared to PR without conical-PEP.

Similar to dyspnea, HRQoL, as measured by the SGRQ, showed statically significant and clinically significant [28] improvements in both groups after 8 weeks of PR. However, no significant difference was found between the two groups. Therefore, the addition of conical-PEP during exercise training to the PR program did not prove to be superior in improving HRQoL in COPD patients. As for the CAT score, both groups' participants had a score of only around 6, indicating low symptoms [29]. Therefore, it is not surprising that there was no significant difference in CAT scores within each group after the PR program, as the low initial scores likely led to a flooring effect.

Although both the conical-PEP group and the control group exhibited significant improvements in exercise capacity, dyspnea, and HRQoL compared to baseline, the findings did not reveal any significant advantage of incorporating conical-PEP into the PR program, compared to the control group (PR without conical-PEP). Post-PR, the conical-PEP group did not show a significant reduction in IC following the ESMT, whereas the control group continued to exhibit a significant reduction. Additionally, the post-PR ESMT revealed no significant between-group differences in the end-exercise percentage of predicted maximum heart rate or RPB (Table S1B), which indicates that both groups performed the test with similar levels of effort. This observation indicates the potential role of conical-PEP in delaying or preventing DH development during exercise. However, as IC was a secondary outcome and the sample size was calculated based on exercise capacity, the study may have been too underpowered to detect a statistically significant between-group difference in IC. Since no statistically significant difference was observed between groups, this finding warrants cautious interpretation.

It might be assumed that wearing an oronasal mask with conical-PEP during exercise would reduce participant compliance compared to conventional exercise. However, the exercise characteristics, including frequency and duration (Table 2), did not differ between the conical-PEP and control groups; this indicates that the mask had no negative impact on adherence to the PR program. The use of the conical-PEP mask during exercise was associated with a high level of compliance, with a reported median usage of 97% (IQR: 72%–100%). Thus, the participants were generally able to integrate the device into their rehabilitation sessions.

We acknowledge several limitations to this study. First, we capped the maximum exercise duration at 60 min per day, which may have limited participants who were capable of exercising for longer durations, particularly in the conical-PEP group. Additionally, some participants did not fully adhere to the exercise protocol, despite efforts to promote both groups' adherence through home visits and telephone calls, which required them to exercise until they reached a dyspnea level of 3–4 out of 10 on the modified Borg scale. This may have hindered the potential effects and benefits of conical-PEP and contributed to the nonsignificant differences observed between the groups in most outcomes. The exercise characteristics were based on the participants' self-reported logbooks, which may not have been entirely accurate. For those who did not record the information immediately after exercising, recall bias may have affected data quality. Furthermore, this study was conducted between October 2016 and December 2017, so the pharmacological treatments used may not reflect current COPD management guidelines. Due to the limited prior research on the incorporation of PEP into exercise training, our sample size calculation was based on references that may not be ideal. Specifically, we relied on a study by Casciari et al., who investigated exercise capacity following a PR program incorporating pursed-lip breathing—an instinctive, nondevice form of PEP [18]. While informative, this study is several decades old, and the management of COPD has since evolved. Moreover, its intervention does not closely reflect the device-based approach used in the present trial. Our more recent study Ubosaka-Jones et al. [17] which was conducted in parallel and not available at the time of sample size planning, involved a similar conical-PEP device during a single exercise session. Although exercise duration was reported in terms of medians and IQRs in that study, the sample size calculation was derived from unpublished mean and SD values (12.34 ± 6.54 and 9.35 ± 5.29 min for the conical-PEP and control groups, respectively), which led to a requirement of 64 participants per group. However, since that study focused on a single-session intervention and did not incorporate conical-PEP into an exercise training program, its sample size estimate was not directly applicable to the present trial. Given the reliance on limited and methodologically different references, our study may have been underpowered. Additional limitations include the relatively short duration of the intervention (8–10 weeks), which may not have been sufficient for detecting long-term benefits. Moreover, the majority of participants were classified as GOLD Stage 2–3, which limits their generalizability to severe, exercise-deprived populations. We also did not assess other physiological markers of hyperinflation, such as RV/TLC, that may have provided a more comprehensive evaluation. Finally, phenotyping patients—for example, through CT imaging to identify emphysema-predominant cases—may have offered additional insights into treatment responsiveness. Further studies should seek to overcome these limitations to provide more robust findings and improve the clinical application of conical-PEP in exercise training programs.

## 5. Conclusion

Exercise training in PR, both with and without conical-PEP, resulted in significantly improved exercise capacity, dyspnea, and HRQoL after 8–10 weeks. The addition of conical-PEP to PR provided no additional benefit. Further research is warranted to better define its role in COPD management.

## Figures and Tables

**Table 1:** Participant characteristics.

**Characteristics**		**Control (** **n** = 21**)**		**Conical-PEP (** **n** = 21**)**		**p** **value**
		**n**	**M** **e** **a** **n** ± **SD**	**n**	**M** **e** **a** **n** ± **SD**	
Gender (male/female)^฿^		17/4		18/3		0.679

Age (years)⁣^∗^			67.2 ± 8.0		64.5 ± 6.8	0.243

Body mass index (kg·m^−2^)⁣^∗^			21.6 ± 4.1		21.9 ± 3.9	0.804

FEV_1_/FVC⁣^∗^			0.51 ± 0.09		0.50 ± 0.12	0.755

FEV_1_% predicted⁣^∗^			56.0 ± 13.1		56.2 ± 15.7	0.966

Gold stage^฿^	II	14		14		1.000
	III	7		5		0.495
	IV	—		2		0.147

Treatment duration (years)^#^			4 [2, 10]		4 [2, 10]	0.930

1 year exacerbation event^#^			0 [0, 1]		0 [0, 1]	0.912

CAT score^#^			6 [3, 12]		6 [2, 12]	0.705

ABCD category^฿^	A	12		11		0.757
	B	5		6		0.726
	C	1		2		0.549
	D	3		2		0.634

Smoking status^฿^	Current smoker	3		2		0.634
	Old smoker	14		16		0.496
	Nonsmoker	2		1		0.549
	Secondhand smoker	2		2		1.000
	Pack year (smokers)⁣^∗^		36.1 ± 21.5		32.0 ± 19.9	0.558

Medication^฿^	SABA (inhaler)	16		10		0.057
	Corticosteroids (inhaler)	1		2		0.549
	Anticholinergic + SABA (inhaler)	5		10		0.107
	Corticosteroids + LABA (inhaler)	19		19		1.000
	Theophylline (oral)	16		14		0.496
	Mucolytic (oral)	5		1		0.189
	Montelukast (oral)	—		1		0.313
	Cetirizine hydrochloride (oral)	1		1		1.000
	Amlodipine (oral)	3		3		1.000
	Doxazosin (oral)	—		2		0.147
	Enalapril (oral)	1		1		1.000
	Losartan potassium (oral)	—		1		0.313
	Lacidipine (oral)	1		—		0.313
	Aspirin (oral)	—		1		0.313
	Simvastatin (oral)	1		—		0.313
	Glipizide	—		1		0.313
	Allopurinol (oral)	1		2		0.549
	Colchicine (oral)	1		4		0.153

Comorbidities^฿^	Hypertension	5		7		0.495
	Diabetes mellitus	—		1		0.313
	Dyslipidemia	1		—		0.313
	Gouty	1		4		0.153

Physical activity	Step/day⁣^∗^		6085 ± 3553		6534 ± 4098	0.709
	Activity time/day (hour)⁣^∗^		12.4 ± 1.9		12.1 ± 1.2	0.689
	Active life style^฿^ (≥ 4500 steps/day) (*n*)	13		14		0.747

*Note:* Data are presented by mean ± SD and median [first quartile, third quartile]. Comparison between groups used statistic as follows: by independent *t*-test (∗), by Mann–Whitney *U* test (#) and ^฿^, by two-sample test for proportions. Step/day and activity time/day were 5 days averaged before start RP program.

Abbreviations: CAT, COPD Assessment Test; FEV1, force expiratory volume in 1 s; FVC, force vital capacity; LABA, long-acting beta 2 agonists; SABA, short-acting beta 2 agonists.

**Table 2:** Exercise characteristics in the pulmonary rehabilitation program.

**Exercise characteristics**	**Control (** **n** = 21**)**	**Conical-PEP (** **n** = 19**)**	**p** **value**
Exercise program			
Program duration (day)^#^	57 [56, 63]	58 [57, 69]	0.136
Program duration (week)^#^	8.1 [8.0, 9.0]	8.3 [8.1, 9.9]	
Exercise day (day)⁣^∗^	40.2 ± 17.8	39.8 ± 15.4	0.929
% exercise day⁣^∗^	66.0 ± 28.4	63.8 ± 25.1	0.792
Exercise time/day (min)∗	28.0 ± 13.1	27.9 ± 7.9	0.981
Exercise frequency (day/week)⁣^∗^	4.6 ± 2.0	4.5 ± 1.8	0.792
Exercise volume (min/week)⁣^∗^	136.7 ± 79.7	124.9 ± 64.6	0.611
Estimated total step in PR (steps)⁣^∗^	110,217 ± 71,932	105,732 ± 48,371	0.820
CPEP-mask usage (%)	—	97 (72, 100)	—
Spot marching exercise (total analysis)			
Exercise time/session(min)^#^	14.8 [5.0, 15.7]	13.5 [9.2, 26.6]	0.323
Session/day (time)^#^	2.0 [1.4, 5.0]	2.0 [1.3, 3.0]	0.615
Exercise step rates (steps/min)∗	88.0 ± 11.7	95.4 ± 14.7	0.076
Adjust by exercise step rates (±SE)			
Exercise time/session (min)^฿^	14.1 ± 2.1	14.6 ± 2.3	0.883
Session/day (time)^฿^	2.9 ± 0.4	2.5 ± 0.5	0.548
Exercise day (day)^฿^	39.2 ± 3.7	41.7 ± 4.1	0.678
% exercise day^฿^	64.5 ± 6.0	66.5 ± 6.7	0.828
Exercise time/day^฿^	28.6 ± 2.5	26.8 ± 2.7	0.637
Exercise frequency (day/week)^฿^	4.5 ± 0.4	4.7 ± 0.5	0.828
Exercise volume (min/week)^฿^	135.5 ± 16.5	127.1 ± 18.2	0.744
Estimated total step in PR (steps)^฿^	112,401 ± 13,972	101,830 ± 15,394	0.628
Termination characteristics in each session of spot marching exercise (*n*)			
Follow instruction:	8	10	0.533
Terminated by RPB 3–4/10^$^			
Nonfollow instruction:			
Self-limited duration^$^	9	6	0.334
(Did not achieve RPB 3–4/10)			
Combine self-limited duration and RPB^$^	2	3	0.634
Nonexercise^$^	1	—	0.312
Distribution of RPB termination exercise time (Achieved RPB 3–4/10, follow instruction)			
< 10 min/session	9	8	
10–< 15 min/session	3	4	
15–< 20 min/session	2	3	
≥ 20 min/session	4	8	
Distribution of self-limited duration (Did not achieve RPB 3–4/10, nonfollow instruction)			
5 min/session	4	6	
10 min/session	3	5	
15 min/session	7	5	
≥ 20 min/session	4	4	

*Note:* Data are mean ± SD and median [first quartile, third quartile]. Two subjects in the CPEP group could not follow at post-PR program (8 weeks); therefore, this report shows summary of 19 subjects in the CPEP-mask group. Estimated total step in PR was calculated from (self-step rate × mean exercise time/day) × exercise day. Comparison between groups used statistics as follows: by independent *t*-test (∗), by Mann–Whitney *U* test (#), by ANCOVA (฿), and by two-sample test of proportion ($).

**Table 3:** Six-minute walk distance and spot marching endurance time at baseline and post-PR.

**Parameters**	**Time**	**Control (** **n** = 21**)**	**Conical-PEP (** **n** = 21**)**	**Between group by time, conical-PEP minus control**		
		**M** **e** **a** **n** ± **SD**	**M** **e** **a** **n** ± **SD**	**Diff**	**95% CI**	**p**
6MWD (m)						
Raw data^$^	Baseline	455.5 ± 97.0	465.9 ± 81.8	10.4	−45.6 to 66.4	0.709
	Post PR	467.7 ± 86.5	498.0 ± 79.7^∗^	30.3	−21.6 to 82.2	0.244
Adjusted by baseline		—	—			
(Mean ± SE)^#^	Post PR	471.8 ± 9.7	494.0 ± 9.7	22.1	−5.7 to 50.0	0.116
ESMT endurance time (min)						
Raw data^$^	Baseline	11.2 ± 6.5	11.4 ± 7.0	−0.2	−4.4 to 4.0	0.916
	Post PR	17.2 ± 9.2^∗∗∗^	16.8 ± 8.4^∗∗∗^	0.4	−5.1 to 5.9	0.877
Step rate (step/min)^$^		88.8 ± 12.8	94.8 ± 15.8	−6.0	−15.0 to 2.96	0.184
Adjusted by step rate						
(Mean ± SE)^#^	Baseline	12.0 ± 1.2	10.5 ± 1.2	−1.5	−5.0 to 2.0	0.389
	Post PR	18.5 ± 1.4	15.5 ± 1.4	−3.0	−7.1 to 1.1	0.149
Adjusted by baseline and step rate						
(Mean ± SE)^#^	Post PR	18.1 ± 1.3	15.9 ± 1.3	−2.2	−5.9 to 1.6	0.247

*Note:* Between-group comparison; $, using independent *t*-test, #, using ANCOVA.

⁣^∗^Significant with baseline ⁣^∗^*p* < 0.05, ⁣^∗∗^*p* < 0.01, and ⁣^∗∗∗^*p* < 0.001, by paired *t*-test.

**Table 4:** Health-related quality of life at baseline and post-PR program.

**Parameters**	**Time**	**Control (** **n** = 21**)**		**Conical-PEP (** **n** = 21**)**		**Diff between groups**
		**Median**	**[Q1, Q3]**	**Median**	**[Q1, Q3]**	
CAT score						
	Baseline	6	[3, 12]	6	[2, 12]	0.705
	Post PR	4	[2, 7]	5	[1, 9]	0.704

SGRQ						

Total score	Baseline	25.6	[18.5, 36.4]	30.7	[14.3, 38.4]	0.811
	Post PR	10.1⁣^∗^	[4.0, 19.5]	11.7⁣^∗^	[3.5, 29.5]	0.687
	Change score	−11.0	[−22.2, −8.5]	−8.2	[−21.5, −0.3]	0.414

Symptom	Baseline	32.8	[17.1, 56.4]	39.2	[23.5, 47.2]	0.753
	Post PR	18.4⁣^∗^	[10.3, 27.8]	21.0⁣^∗^	[9.2, 29.8]	0.791

Activity	Baseline	22.3	[14.8, 51.7]	44.9	[15.4, 52.1]	0.521
	Post PR	7.4⁣^∗^	[0.0, 29.7]	14.9⁣^∗^	[0.0, 37.6]	0.442

Impact	Baseline	25.7	[11.8, 37.5]	24.7	[9.7, 31.2]	0.624
	Post PR	4.8⁣^∗^	[0.0, 12.2]	6.6⁣^∗^	[0.0, 16.0]	0.609

*Note:* Between-group comparison used Mann–Whitney *U* test. Within-group comparison used Wilcoxon signed-rank test.

Abbreviations: CAT, COPD Assessment Test; SGRQ, St. George's Respiratory Questionnaire.

⁣^∗^*p* < 0.05 between baseline versus post PR.

**Table 5:** Lung function at baseline and post-PR program.

**Parameters**	**Time**	**Control (** **n** = 21**)**	**Conical-PEP (** **n** = 21**)**	**p** **value**	**Total (** **n** = 42**)**
FVC (L)	Baseline	2.59 ± 0.80	2.82 ± 0.73	0.340	2.71 ± 0.77
	Post PR	2.64 ± 0.82	2.82 ± 0.74	0.457	2.73 ± 0.78
FVC %predict	Baseline	76.3 ± 14.4	78.4 ± 12.4	0.615	77.3 ± 13.3
	Post PR	77.0 ± 16.2	78.6 ± 13.0	0.715	77.8 ± 14.5
FEV_1_ (L)	Baseline	1.32 ± 0.44	1.41 ± 0.42	0.499	1.36 ± 0.42
	Post PR	1.40 ± 0.47^⸸⸸^	1.43 ± 0.46	0.855	1.41 ± 0.46^⸸⸸^
FEV_1_ %predict	Baseline	56.0 ± 13.1	56.2 ± 15.7	0.966	55.1 ± 13.4
	Post PR	59.1 ± 14.6^⸸^	57.3 ± 16.9	0.706	58.2 ± 15.7^⸸^
FEV_1_/FVC %	Baseline	51.2 ± 8.8	50.2 ± 12.4	0.755	50.7 ± 10.7
	Post PR	53.7 ± 9.5	50.8 ± 13.0	0.429	52.2 ± 11.3^⸸^
FEF_25–75_ (L/s)	Baseline	0.51 ± 0.25	0.56 ± 0.31	0.555	0.53 ± 0.28
	Post PR	0.59 ± 0.28	0.59 ± 0.31	0.992	0.59 ± 0.29^⸸⸸^
FEF_25–75_ %predict	Baseline	20.6 ± 7.6	21.8 ± 10.7	0.668	21.2 ± 9.2
	Post PR	23.3 ± 9.4^⸸^	22.8 ± 11.7	0.874	24.1 ± 11.7^⸸^
PEF (L/s)	Baseline	3.56 ± 1.76	4.12 ± 1.43	0.262	3.84 ± 1.61
	Post PR	4.40 ± 1.57^⸸^	4.61 ± 1.54^⸸^	0.663	4.51 ± 1.54^⸸⸸^
PEF %predict	Baseline	51.8 ± 18.9	55.5 ± 17.7	0.509	53.6 ± 18.2
	Post PR	59.6 ± 20.6^⸸^	61.0 ± 19.0^⸸^	0.823	60 3 ± 19.6^⸸⸸^
MIP (cm H_2_O)	Baseline	86.2 ± 35.0	88.6 ± 33.4	0.822	87.4 ± 33.8
	Post PR	92.1 ± 33.6	94.0 ± 30.8	0.849	93.9 ± 31.7
MEP (cm H_2_O)	Baseline	104.2 ± 31.1	117.8 ± 46.6	0.282	111.1 ± 39.9
	Post PR	110.1 ± 26.0	119.4 ± 36.6	0.349	115.3 ± 31.9

*Note:* Data are mean ± SD. Comparison between groups used independent *t*-test. Significant differences within the group (baseline vs. post-PR) were shown by ^⸸^*p* < 0.05, ^⸸⸸^*p* < 0.01, using the paired *t*-test.

Abbreviations: FEF, force expiratory flow; FEV_1_, force expiratory volume in 1 s; FVC, force vital capacity; MEP, maximum expiratory pressure; MIP, maximum inspiratory pressure; PEF, peak expiratory flow.

## Data Availability

Data related to this study are available from the corresponding author upon reasonable request.

## Supporting Information

Supporting Information Additional supporting information can be found online in the Supporting Information section. Table S1A: Pre-exercise and end-exercise cardiopulmonary responses, breathlessness, and leg fatigue in the 6MWT at baseline and post-PR program. Table S1B: Pre-exercise and end-exercise cardiopulmonary responses, breathlessness, and leg fatigue in the ESMT at baseline and post-PR program.
