# Supplementary material for: Effects of Conical Positive Expiratory Pressure Mask Application During Exercise Training on Pulmonary Rehabilitation Outcomes in Moderate to Severe COPD Cases: A Randomized Controlled Trial
Source: Pulm Med. 2025 Oct 31;2025:8828987. doi: 10.1155/pm/8828987 (PMC12595236; doi:10.1155/pm/8828987)
Supplement: Supporting Information — Additional supporting information can be found online in the Supporting Information section. Table S1A: Pre-exercise and end-exercise cardiopulmonary responses, breathlessness, and leg fatigue in the 6MWT at baseline and post-PR program. Table S1B: Pre-exercise and end-exercise cardiopulmonary responses, breathlessness, and leg fatigue in the ESMT at baseline and post-PR program. [file 8828987.f1.docx]

**Table Supplement 1A** Cardiopulmonary responses, breathlessness and leg fatigue of 6MWT at pre and end-exercise in baseline and post PR program

| **Parameters** | **Times** | **Pre-exercise** | | |  | **End-exercise** | | |  |
| --- | --- | --- | --- | --- | --- | --- | --- | --- | --- |
|  |  | **Control (n=21)** | **Conical-PEP (n=21)** | **p-value** |  | **Control (n=21)** | **Conical-PEP (n=21)** | **p-value** |  |
| RR (breath/min)* | Baseline | 21 ± 4 | 23 ± 6 | 0.220 |  | 25 ± 5 | 25 ± 4 | 0.975 |  |
|  | Post PR | 21 ± 4 | 22 ± 5 | 0.356 |  | 26 ± 7 | 25 ± 6 | 0.794 |  |
| P_et_CO_2_ (mm Hg)* | Baseline | 31.9 ± 4.9 | 32.5 ± 4.5 | 0.685 |  | 37.8 ± 5.8 | 39.4 ± 6.7 | 0.415 |  |
|  | Post PR | 32.1 ± 4.2 | 31.2 ± 5.0 | 0.507 |  | 38.9 ± 6.7 | 39.1 ± 6.1 | 0.886 |  |
| S_p_O_2_ (%)^#^ | Baseline | 96 [95 , 97] | 97 [95 , 97] | 0.507 |  | 96 [95 , 97] | 96 [94 , 98] | 0.950 |  |
|  | Post PR | 96 [96 , 98] | 96 [95 , 98] | 0.761 |  | 95 [95 , 98] | 95 [91 , 98] | 0.491 |  |
| Borg’s RPB^#^ | Baseline | 0 [0 , 0] | 0 [0 , 0] | 0.317 |  | 2 [0.5 , 3] | 2 [1 , 3.5] | 0.518 |  |
|  | Post PR | 0 [0 , 0] | 0 [0 , 0] | 0.317 |  | 1 [0 , 2] | 2.5 [1 , 3.5] | 0.062 |  |
| Borg’s Leg fatigue^#^ | Baseline | 0 [0 , 0] | 0 [0 , 0] | 0.593 |  | 0 [0 , 2] | 1 [0 , 2] | 0.216 |  |
|  | Post PR | 0 [0 , 0] | 0 [0 , 0] | 0.317 |  | 0.5 [0 , 2] | 1 [0 , 2] | 0.817 |  |
| HR (bpm)* | | Baseline | 89 ± 13 | 85 ± 15 | 0.306 |  | 115 ± 17 | 111 ± 17 | 0.506 |
|  | | Post PR | 84 ± 11 | 83 ± 14 | 0.831 |  | 109 ± 14 | 111 ± 14 | 0.764 |
| %maxHR* | | Baseline | 55.5 ± 7.1 | 52.1 ± 8.9 | 0.187 |  | 71.2 ± 9.2 | 68.4 ± 10.8 | 0.367 |
|  | | Post PR | 52.2 ± 6.3 | 51.2 ± 8.1 | 0.634 |  | 68.0 ± 7.6 | 68.2 ± 8.4 | 0.960 |
| SBP (mm Hg)* | | Baseline | 125.0 ± 18.7 | 122.0 ± 15.9 | 0.576 |  | 145.8 ± 23.8 | 151.2 ± 18.6 | 0.417 |
|  | | Post PR | 118.4 ± 17.0 | 119.4 ± 13.6 | 0.846 |  | 143.4 ± 19.5 | 149.8 ± 23.7 | 0.347 |
| DBP (mm Hg)* | | Baseline | 78.9 ± 8.3 | 77.7 ± 7.9 | 0.621 |  | 87.4 ± 12.5 | 84.3 ± 11.6 | 0.415 |
|  | | Post PR | 75.2 ± 8.3 | 76.5 ± 8.0 | 0.632 |  | 78.9 ± 10.3 | 82.2 ± 16.6 | 0.440 |
| MAP (mm Hg)* | | Baseline | 94.3 ± 12.6 | 88.4 ± 13.1 | 0.150 |  | 108.3 ± 17.8 | 104.9 ± 16.6 | 0.525 |
|  | | Post PR | 89.1 ± 10.8 | 88.3 ± 9.7 | 0.794 |  | 103.7 ± 16.2 | 102.0 ± 16.6 | 0.744 |

Note. Data are mean ± SD and median [Q1,Q3], ex; exercise, comparison between groups at end-exercise using independent t-test,
RR; respiratory rate, P_et_CO_2_; end tidal carbon dioxide, SpO_2_; pulse oxygen saturation, RPB; breathlessness, HR; heart rate, %maxHR; percentage of age related maximum heart rate, SBP; systolic blood pressure, DBP; diastolic blood pressure, MAP; mean arterial pressure. * by independent t-test, # by Mann Whitney U test.

**Table Supplement 1B** Cardiopulmonary responses, breathlessness and leg fatigue of ESMT at pre and end-exercise in baseline and post PR program

| **Parameters** | **Times** | **Pre-exercise** | | |  | **End-exercise** | | |
| --- | --- | --- | --- | --- | --- | --- | --- | --- |
|  |  | **Control (n=21)** | **Conical-PEP (n=21)** | **p-value** |  | **Control (n=21)** | **Conical-PEP (n=21)** | **p-value** |
| RR (breath/min)* | Baseline | 20 ± 4 | 21 ± 3 | 0.901 |  | 30 ± 7 | 31 ± 6 | 0.808 |
|  | Post PR | 20 ± 4 | 21 ± 4 | 0.521 |  | 30 ± 7 | 29 ± 6 | 0.422 |
| P_et_CO_2_ (mm Hg)* | Baseline | 30.3 ± 4.1 | 31.1 ± 4.0 | 0.523 |  | 34.9 ± 5.0 | 35.1 ± 5.3 | 0.859 |
|  | Post PR | 30.8 ± 4.0 | 30.5 ± 4.5 | 0.773 |  | 33.5 ± 3.9 | 34.2 ± 6.1 | 0.634 |
| S_p_O_2_ (%)^#^ | Baseline | 97 [96 , 99] | 97 [96 , 99] | 0.659 |  | 95 (94 , 97] | 96 [92 , 98] | 0.781 |
|  | Post PR | 98 [96 , 98] | 97 [96 , 98] | 0.620 |  | 96 (95 , 98] | 95 [93 , 98] | 0.291 |
| Borg’s RPB^#^ | Baseline | 0 [0 , 0] | 0 [0 , 0] | 1.000 |  | 5 [3 , 6.5] | 5 [3 , 6] | 0.859 |
|  | Post PR | 0 [0 , 0] | 0 [0 , 0] | 1.000 |  | 3 [0.5 , 5] | 5 [1 , 6.5] | 0.306 |
| Borg’s Leg fatigue^#^ | Baseline | 0 [0 , 0] | 0 [0 , 0] | 0.576 |  | 5 [3 , 6.5] | 5 [4 , 5.5] | 0.859 |
|  | Post PR | 0 [0 , 0] | 0 [0 , 0] | 0.317 |  | 3 [0 , 6] | 4.5 [1 , 6.5] | 0.306 |
| HR (bpm)* | Baseline | 84 ± 12 | 83 ± 13 | 0.826 |  | 114 ± 19 | 112 ± 15 | 0.677 |
|  | Post PR | 79 ± 10 | 80 ± 13 | 0.780 |  | 107 ± 16. | 106 ± 16 | 0.855 |
| %maxHR* | Baseline | 51.9 ± 7.5 | 50.4 ± 7.6 | 0.832 |  | 70.7 ± 11.9 | 69.4 ± 9.1 | 0.704 |
|  | Post PR | 48.7 ± 6.4 | 49.3 ± 7.2 | 0.762 |  | 66.4 ± 10.3 | 66.0 ± 10.0 | 0.896 |
| SBP (mm Hg)* | Baseline | 120.2 ± 14.4 | 119.5 ± 15.5 | 0.886 |  | 141.1 ± 15.8 | 140.6 ± 23.0 | 0.932 |
|  | Post PR | 121.3 ± 18.4 | 118.4 ± 16.2 | 0.596 |  | 137.6 ± 20.4 | 142.3 ± 22.3 | 0.483 |
| DBP (mm Hg)* | Baseline | 77.3 ± 6.3 | 78.0 ± 7.6 | 0.733 |  | 80.0 ± 10.8 | 78.0 ± 9.6 | 0.511 |
|  | Post PR | 78.4 ± 11.4 | 77.8 ± 8.4 | 0.854 |  | 80.1 ± 11.5 | 82.4 ± 16.8 | 0.609 |
| MAP (mm Hg)* | Baseline | 90.5 ± 8.5 | 89.5 ± 10.1 | 0.737 |  | 102.8 ± 14.1 | 98.4 ± 19.1**^⸸^** | 0.406 |
|  | Post PR | 90.1 ± 13.5 | 89.1 ± 13.0 | 0.800 |  | 98.8 ± 15.4 | 101.7± 21.6 | 0.620 |

Note. Data are mean ± SD and median [Q1,Q3], ex; exercise, comparison between groups at end-exercise using independent t-test,
RR; respiratory rate, P_et_CO_2_; end tidal carbon dioxide, SpO_2_; pulse oxygen saturation, RPB; breathlessness, HR; heart rate, %maxHR; percentage of age related maximum heart rate, SBP; systolic blood pressure, DBP; diastolic blood pressure, MAP; mean arterial pressure. * by independent t-test, # by Mann Whitney U test.
